# Shared Psychophysiological Electroencephalographic Features in Maltreated Adolescent Siblings and Twins: A Case Report

**DOI:** 10.7759/cureus.63269

**Published:** 2024-06-27

**Authors:** Gabriela M Marcu, Raluca-Diana Szekely-Copîndean, Ana-Maria Zăgrean

**Affiliations:** 1 Department of Functional Sciences, Division of Physiology II - Neuroscience, Carol Davila University of Medicine and Pharmacy, Bucharest, ROU; 2 Department of Psychology, Lucian Blaga University of Sibiu, Sibiu, ROU; 3 Department of Social and Human Research, Romanian Academy, Cluj-Napoca Branch, Cluj-Napoca, ROU

**Keywords:** quantitative electroencephalography, adolescents, complex trauma, childhood maltreatment, event-related potentials, electroencephalography (eeg)

## Abstract

This case report presents a comprehensive assessment of four maltreated adolescents, two half-siblings, and two non-identical twins to investigate the effects of complex childhood trauma on brain functioning. The study aimed to identify shared psychophysiological features in the electroencephalographic (EEG) data of these adolescents compared to database norms. Quantitative EEG, event-related potentials (ERPs), and their independent components were analyzed to examine alterations in patterns of electrical activity associated with psychopathology. In the half-sibling pair, enhanced P1 and N1 amplitudes were observed during the cued Go/NoGo task, while reduced N2 amplitude was present in the fraternal twins. The type of trauma also seems to affect EEG spectral distribution and higher-order cognitive processes, such as attention allocation and response inhibition (N2 wave). Specifically, physically abused and bullied adolescents showed reduced N2 amplitudes and lower alpha power in the posterior region. No significant differences were noted in the ERP-independent components for maltreated adolescents compared to norms. The analysis of these cases aimed to provide insights into the neurobiological substrates underlying the overlapping symptoms and syndromes of child maltreatment, which may aid in differential diagnosis and the development of targeted interventions for trauma-related psychopathology in adolescents.

## Introduction

Trauma-related psychopathology remains a puzzle that needs to be understood in the presence of a high need for efficient interventions. Some primarily related conditions include post-traumatic stress disorder (PTSD), complex PTSD (CPTSD), dissociative disorders, borderline personality disorder (BPD), substance use disorders, depression, and anxiety. While there is abundant research on the impact of traumatic experiences on adults, particularly those related to PTSD, our understanding of how these experiences affect individuals at varying developmental stages, such as children and adolescents, is limited. This field is particularly challenging to study because, during these critical stages, the brain, which is most affected by trauma exposure, is still developing, and in many cases, trauma exposure is still ongoing. Trauma exposure during childhood has severe developmental consequences (like impaired attachment and trust issues, emotional dysregulation, dissociation, and behavioral control problems), which can lead to deficits and delays in major developmental milestones across various biological structures (prefrontal cortex, limbic structures, and hypothalamic-pituitary-adrenal (HPA) axis), contributing to comorbid psychopathology [1,2].

In their proposed theoretical model, McLaughlin et al. [3] identified two dimensions of adverse childhood experiences (ACEs): threat and deprivation, each with a different impact on the psychophysiological development of the child. The threat includes physical abuse and often leads to difficulties in emotional reactivity and emotion regulation, while deprivation involves neglect and has been associated with cognitive alterations such as language, executive functioning, and complex problem-solving [4]. The concept of CPTSD has been proposed to capture both the distinct aspects of traumatic exposure complexity and typical symptoms of PTSD. Consequently, new clusters, such as identity and relational disturbances, have been considered for the diagnosis of CPTSD. The term “complex” illustrates the exposure to multiple, longstanding, and often interrelated forms of traumatic experiences, leading to a wide range of psychiatric diagnoses and misdiagnoses, functional impairments, and evolving educational, vocational, relational, and health problems. These include frequent misdiagnoses of mood disorders, attention-deficit/hyperactivity disorder (ADHD), oppositional defiant disorder, conduct disorder, sleep disorders, reactive attachment disorder, and personality disorders due to the overlap of trauma-related symptoms [1].

Adolescence is the peak time for developing brain areas responsible for the domains of executive functioning (self-awareness, cognitive control, and self-reference). Executive functions are essential for independent functioning and healthy relationships [1]. Neuroscientific research suggests that subtle deficits in attention regulation and response inhibition may precede the development of PTSD symptoms and, as risk factors, may be related to symptom severity [5]. Research has suggested that early traumatic experiences affect both cortical and subcortical areas of the brain, resulting in frontal lobe abnormalities, temporal-posterior altered alpha activity, and an overactive limbic system [1,6,7].

Current diagnostic criteria for PTSD/CPTSD may not adequately identify how children and adolescents express symptoms in response to maltreatment. A developmental trauma (DT) framework was proposed to offer a broader perspective, to highlight the importance of considering symptoms as reactions to trauma rather than separate mental health issues, and to describe “children who have been traumatically victimized and whose attachment bonding with primary caregivers has been compromised” [8]. DT was often associated with trait anxiety, a personality characteristic that reflects a stable tendency to respond to anxiety in the face of perceived threats. For example, children who experience DT were described as having a higher risk of developing a heightened sensitivity to stress, leading to trait anxiety [6], while childhood emotional abuse and neglect were associated with more severe social anxiety and trait anxiety in adulthood [9]. Therefore, it is important to identify the phenotypic features central to the overlapping symptoms and syndromes of child maltreatment, which may indicate common neurobiological substrates. This will help determine the clinical presentations for differential diagnosis, particularly fear-related symptoms.

In this case analysis, our research question was whether certain psychophysiological features of brain functioning (quantitative EEG, event-related potentials (ERPs), and their independent components) of foster-care maltreated adolescents would exhibit alterations in patterns of electrical activity compared to database norms. EEG gives a broad view of brain activity, while qEEG, also called brain mapping, uses computerized analysis of EEG data to provide a more detailed and quantitative analysis. ERPs specifically focus on how the brain responds to certain stimuli or events. The last two (qEEG and ERPs) are more informative when looking for abnormal patterns or responses as they provide objective, quantifiable data on brain function, aiding in the diagnosis, understanding, and treatment. For example, for psychological trauma, qEEG may enable the identification of specific brain regions affected by trauma and further guide some personalized treatment approaches, such as neurofeedback therapy, by comparing an individual's brain activity to normative databases. ERPs are particularly useful for assessing cognitive and emotional processing in trauma-exposed individuals, as they can reveal how the brain responds to trauma-related stimuli and help identify altered neural pathways associated with trauma.

Changes observed in ERPs and their latent cognitive components were examined in association with psychopathology. To gain a more comprehensive understanding of the variations in neural responses, we examined anxiety- and conflict-related ERPs originating from two brain processing systems. (1) Sensory system: Because traumatic experiences can affect early sensory processing, leading to changes in the P1 and N1 components, individuals with traumatic exposure may exhibit enhanced P1 and N1 amplitudes when exposed to trauma-related stimuli or only when dealing with a task that elevates anxiety, which we found in the pair of half-siblings. (2) Cognitive system: Trauma can affect higher-order cognitive processes, such as attention allocation and response inhibition (N2 wave), which can lead to reduced N2 amplitude during tasks involving conflict monitoring or error detection as well as poor performance in Go trials.

To gain a deeper understanding of the effects of maltreatment/complex childhood trauma (CCT) on adolescents, we analyzed two pairs of siblings (12-17 years old). One pair had only a maternal lineage, whereas the other were fraternal twins. Both pairs shared the same familial environment until they entered the protection system, where they continued to have similar environmental settings. We included foster care maltreated adolescents due to their similar experience of trauma from abandonment and interpersonal trauma suffered while in care. They also scored more than 6 on the ACEs inventory, meaning the adolescents suffered from more than six adversities during childhood. Our comprehensive analysis included demographics, psychopathology, and physiological brain assessments (psychophysiology).

## Case presentation

We present a comprehensive assessment involving demographic, trauma, and psychological evaluations of four maltreated adolescents, consisting of two half-siblings and two non-identical twins. The demographic information and trauma for each adolescent are shown in Table 1. All evaluation instruments are described in the preregistered protocol for data collection [10].

**Table 1:** Demographic information and trauma assessment scores

Group	Half-siblings		Twins	
ID	S1	S2	T1	T2
Age (years)	13	17	15	15
Sex	female	female	female	male
IQ	92	72	85	100
ACEs (adverse childhood experiences)	6	6	11	10
PTSD (post-traumatic stress disorder)	6	14	10	14
DSO (disturbances in self-organization)	0	3	12	12
CPTSD (complex post-traumatic stress disorder)	6	17	22	26
Subjective symptoms evaluation	21	38	29	35

Half-siblings

The two half-siblings come from a family of seven siblings, four of whom have been placed in foster care. The family is struggling financially; the mother is unemployed and frequently leaves the house, while the father is an alcoholic and displays aggressive behavior. He is the biological father of the 17-year-old girl. At the time of data collection, the mother had left home and could not be reached, so the children had lost contact with her.

Sibling 1 (S1) is 13 years old and entered the protection system when she was eight because of neglect and exploitation. Exploitation refers to making her perform labor beyond her physical capability when she should be attending school instead of working. She has a psychiatric diagnosis for emotional childhood disturbances and presents cognitive deficiencies as well as affective regulation problems. Despite these challenges, she has made good progress and is cooperative and affectionate in her foster home. S1 performs well in school, is compliant, well-behaved, and has good social connections with both peers and adults, as assessed by the center’s psychologist. Sibling 2 (S2), 17 years old, entered the protection system at the age of 10 because of neglect, physical abuse, and exploitation. She suffered from severe neglect and was consistently physically assaulted by her biological father. She was diagnosed with emotional childhood disturbance. S2 is shy, has low self-esteem, is insecure, and sometimes acts impulsively. She is also involved in risky peer relationships and behaviors, such as running away and alcohol consumption, and she was also the victim of more physical attacks and bullying while in foster care. She experienced high anxiety and occasional panic attacks. S2 is rather uncommunicative and has a low tolerance for frustration, but she also demonstrates good self-care in her physical appearance.

Twins

The 15-year-old twins come from a family with six siblings from different fathers. They used to live with their mother and partner after their biological father left because of allegations of sexual assault. In their mother's home, they experienced constant physical abuse and neglect until they were taken into foster care by the protection system at the age of nine. Both have been previously diagnosed with ADHD.

As assessed by the center’s psychologist, Twin 1 (T1) is communicative and shows good social skills. She also exhibits difficulties in conflicting situations, emotional instability, and some learning difficulties. Twin 2 (T2) is somewhat reserved for communication and occasionally struggles to express thoughts. The psychologist's evaluation revealed impulsiveness, emotional instability, insecurity, relationship difficulties, and a tendency toward isolation. T2 often experiences exclusion within the children's group at the center, with instances of bullying that include making mean remarks and using labels.

Psychological and symptoms assessment

**Figure 1:**
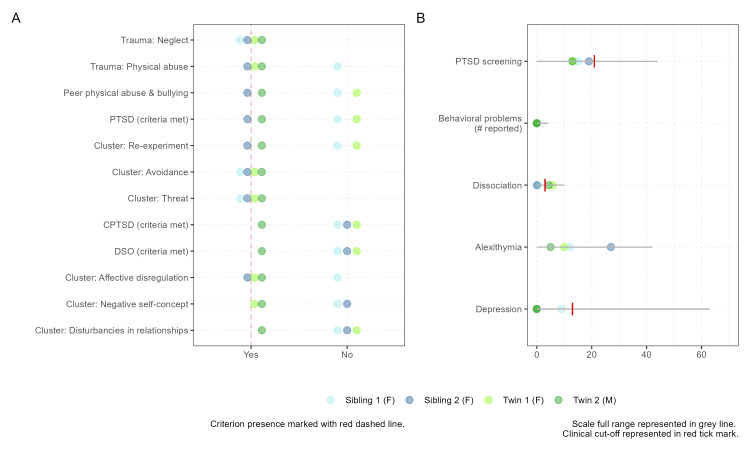
Psychological assessment of maltreated half-siblings (blue) and twin-siblings (green) Panel A: Graphical representation of trauma classification (type of trauma - first two rows - and diagnostic criteria for clusters of CPTSD - rows 3-11). Panel B: Quantitative results of psychopathology assessment. Individual scores and cutoff scores (S1, S2, T1, T2, cutoff): PTSD screening (UCLA-Ri): 15, 19, 13, 13, 21; Alexithymia (CAM): 12, 27, 10, 5, N/A, Depression (BDI II): 9, 0, 0, 0, 13; Dissociation (DES): 0.1, 0.07, 5.8, 4.37, 4. CPTSD: complex post-traumatic stress disorder; PTSD: post-traumatic stress disorder; UCLA-Ri: University of California, Los Angeles - Reaction Index; CAM: Children’s Alexithymia Measure; BDI II: Beck Depression Inventory II; DES: Dissociative Experiences Scale; DSO: disturbances in self-organization

The maltreatment type and psychological symptoms evaluation are detailed in Figure 1. The four adolescents exhibit common characteristics including severe neglect, more than six ACEs, clusters of Avoidance and Sense of Threat, and no (except one) clinical scores on tests and scales for PTSD screening (University of California, Los Angeles - Reaction Index (UCLA-Ri)), complex trauma assessment (International Trauma Questionnaire - Child and Adolescent (ITQ-CA)), depression (Beck Depression Inventory II (BDI II)), and alexithymia (Children’s Alexithymia Measure (CAM)). These shared descriptors form a basis for further comparing EEG-derived features and addressing the limitations of subjective rating-based assessments, which may not capture the full extent of impairments related to the impact of trauma on functionality.

Quantitative EEG assessment

**Figure 2:**
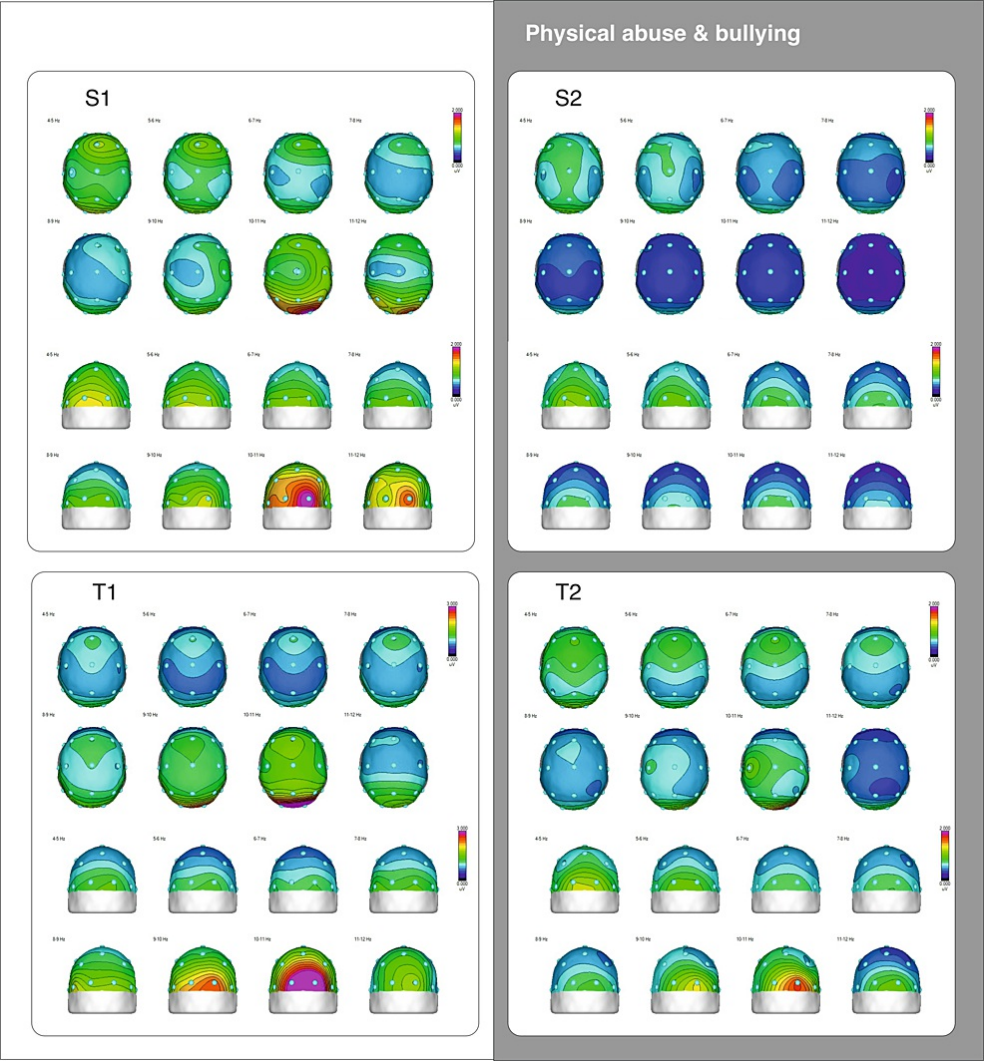
Topographical maps of theta and alpha frequencies distribution in maltreated adolescent pairs The scalp map colors represent brain wave amplitudes in uV at specific frequencies. Red indicates higher amplitudes, while dark blue indicates lower amplitudes. The half-maps display the back of the brain, with the occipital sites (O1 and O2) centrally, to reveal the location of the highest alpha amplitude. S1-S2: half-siblings; T1-T2: twins

EEG data were collected using a 19-channel Mitsar-EEG-BT Lite QEEG edition system (Mitsar Co., Ltd., Saint Petersburg, Russia) along with a 21-electrode EEG cap (Medcap; Medical Computer Systems (MCS), Moscow, Russia) with AgCl electrodes placed according to the 10-20 system, as described in the Preregistration for Data Collection. Each participant completed a 20-minute visual cognitive task consisting of 400 trials using a cued Go/NoGo paradigm. Each participant's EEG spectral analysis was compared to the Human Brain Institute (HBI) database [11], and spectral distributions of siblings’ EEG generated during the task were compared in each pair (Figure 2). As measured in our study, during a cognitive visual task, qEEG and EEG measure different frequency bands of brain wave activity, each reflecting specific cognitive processes. Delta waves (0.5-4 Hz) might still be present but are generally minimal, as they are associated with deep sleep and restorative processes. Of interest were theta waves (4-8 Hz), which can indicate focused attention, creativity, and the engagement of working memory during the task, and Alpha waves (8-12 Hz), reflecting reduced relaxation and increased visual processing and cognitive engagement. Alpha wave patterns, particularly alpha desynchronization, play a significant role in the cognitive processes underlying response inhibition and attentional control during the Go/NoGo paradigm. The power of these waves, or their amplitude, shows the intensity of brain activity in each frequency band, revealing how the brain allocates resources to handle the cognitive demands of the visual task.

Compared to the controls, the half-siblings showed significant differences in the spectral power of the theta and beta bands in the central electrodes (Fz, Cz), while in the twin pair, only T1 exhibited significant differences in the beta band (Fz and Pz). The EEG power of all four participants was consistently lower than that of the control group. This supports the qEEG marker for trauma proposed in one of our recent studies, which indicated a decrease in total absolute power across the EEG spectrum (Marcu et al. (2024)). In our previous study on the brain physiology of trauma-exposed adolescents, we found that in the resting-state EEG, there was a decrease in alpha power in posterior locations, lower values of individual alpha peak frequency, higher variability in fronto-lateral and temporo-parietal alpha asymmetry, and lower EEG power in the trauma group. However, only the lower EEG power in the trauma group was statistically significant compared to controls.

When compared within pairs, the half-siblings showed differences in posterior alpha power and frontal theta, with S2 showing lower EEG power in most frequency bands and no visible alpha peak frequency. For the twins, there were differences in posterior alpha power (around 10 Hz) and slow temporal alpha (8.79 Hz), with both twins sharing the same individual alpha peak frequency, but T2 showing a weaker posterior alpha power.

The topoplots in Figure 2 reveal two similarities in the task-related EEG spectral distribution. The twins exhibited similar topographies for the midline theta band, particularly in the 6-8 Hz range. Furthermore, adolescents who experienced physical assault and current bullying (S2 and T2) showed a reduction in upper alpha band power, particularly at the posterior site.

ERPs, latent ERP independent components (ICs), and associated neuropsychological parameters

**Figure 3:**
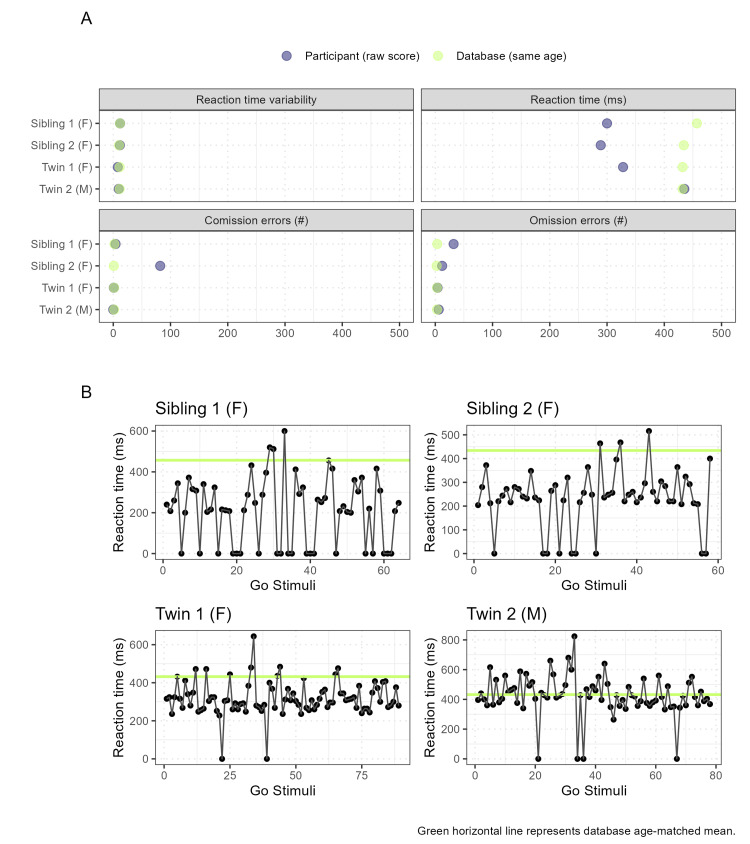
Behavioral results of maltreated siblings (S1, S2) and twins (T1, T2) in Go/NoGo task Panel A: Graphs of reaction time variability, reaction time, omission, and commission error scores (blue dots: participants, green dots: healthy controls mean results from HBI database). Panel B: Reaction time for each participant during the task. The age-matching norm (green) for each adolescent was retrieved from the HBI database. HBI: Human Brain Institute

Among the changes in brain neurophysiology, traumatic disorders such as PTSD or other conditions related to trauma can change the patterns of ERPs [12]. These changes often indicate alterations in how the brain processes information, attention mechanisms, emotional regulation, and cognitive functioning. It is possible that these changes can affect the underlying components of ERPs. We employed the cued Go/NoGo paradigm, which is highly replicable, as one of the tasks most sensitive to cognitive control [13]. The behavioral results are illustrated in Figure 3.

The number of errors and variability in the reaction times of the adolescents were close to the normative database values (Figure 3). Both siblings and T1 had faster reaction times, while T2 had similar reaction times relative to the mean age-matched database norm (Figure 3, Panel A) and throughout the Go/NoGo task span (Figure 3, Panel B). The Go/NoGo paradigm is an experimental measure of response inhibition, but it may not capture the subtle changes in behavior induced by trauma if only observable measures of error and reaction time are used [14]. Therefore, task-generated ERPs, such as conflict-related components, are key windows for examining cognitive control in clinical populations, particularly in trauma-related disorders. For our analysis, we chose three components: one pertains to the sensory system, namely, the P1/N1 response, and has posterior topography. The other two components relate to the cognitive system and reflect unique operations for the NoGo condition: conflict detection and monitoring (N2 response, with frontal topography) and action inhibition (P3 response, with central topography) [13]. The N2/P3 complex is generated in the prefrontal cortex, an area of intense development during adolescence, and is associated with executive functioning, which is crucial for self-regulation. Studies suggest that genetic factors influence response inhibition, as seen in the correlations between siblings and twins during cognitive tasks [15].

**Figure 4:**
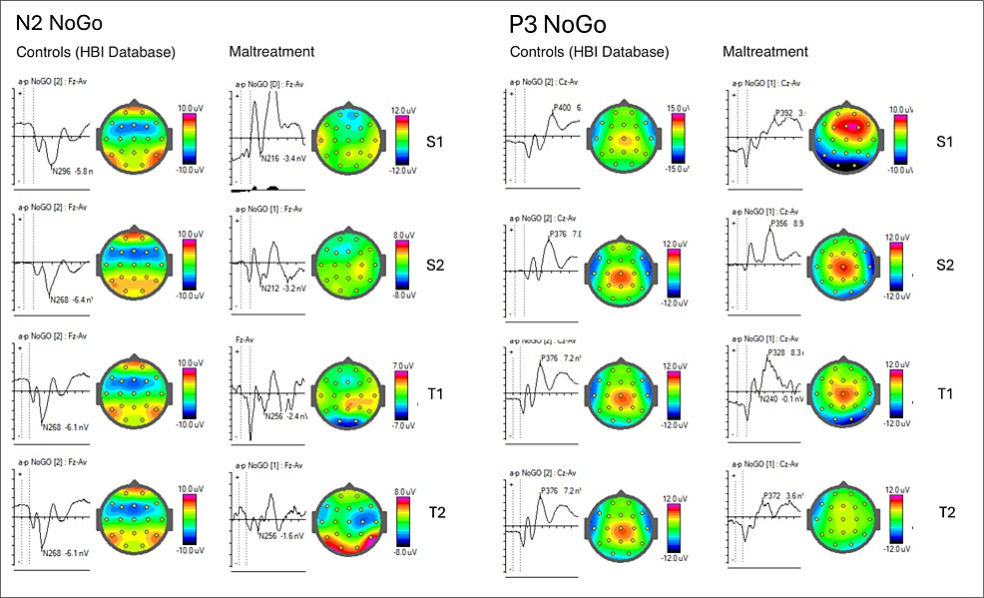
ERPs in NoGo condition (cued Go/GoNo visual task, after the second stimulus), for each participant, compared with the database. Latencies and amplitudes are marked in the wave graphs, and the components' topographies are displayed to the right of each graph (half-siblings (S1-S2) and twins (T1-T2)). ERPs: event-related potentials

**Figure 5:**
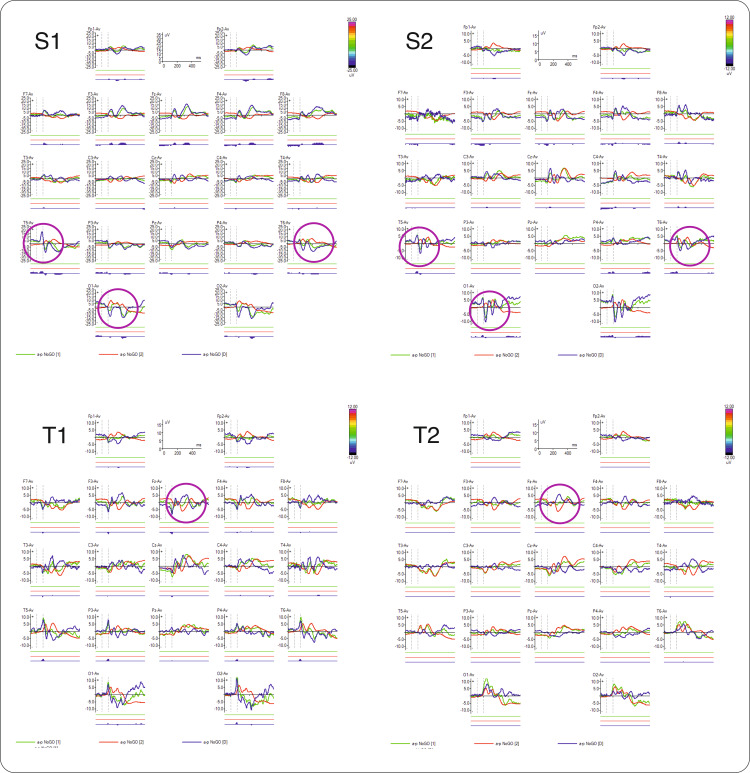
ERPs in NoGo condition (cued Go/GoNo visual task, after the second stimulus), for each case, in all electrode locations. Purple circles highlight pair similarities (half-sibling (S1-S2) and twins (T1-T2)). ERPs: event-related potentials

Therefore, in the NoGo condition, we compared the ERPs with the HBI database (Figure 4) and between siblings in both pairs (Figure 5) to identify similarities. We observed that the half-siblings displayed similarity in early visual components measured in temporal and posterior sites in both hemispheres, while the twins exhibited similarities in the later cognitive component N2 NoGo.

Compared with the control group from the database, all participants had smaller N2 NoGo amplitudes (differences statistically significant only for S2, p<0.02), while the half-siblings also had faster latencies. For the P3 NoGo component, only S1 showed statistical differences in amplitude (p<0.001), while latencies were within the normal window for all participants.

**Figure 6:**
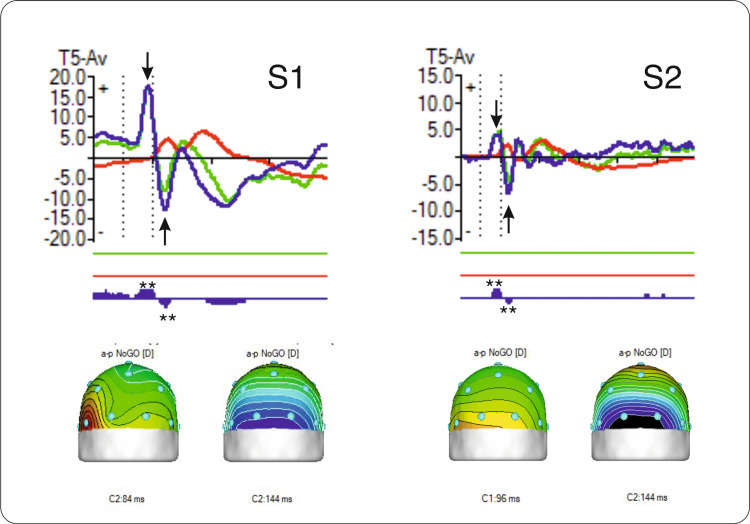
Similarities of N1/P1 complex in half-siblings Top row: N1/P1 complex of early ERP components in NoGo trials in maltreated half-siblings (S1 and S2). Green line = participant; red line = controls from the database; blue line = difference wave. Arrows indicate the component peaks. Double asterisks (**) indicate significant differences from controls (p<0.00) at each peak. Bottom row: Topographical maps of P1 and N1 components' peak location and latency (in milliseconds) for the NoGo trials. ERP: event-related potential

The N1/P1 complex of early components significantly differs from the controls from the HBI database (p<0.001 in S1 and p<0.005 in S2, Figure 6) and may indicate encoding deficits or atypical sensorial reactivity associated with anxiety and repetitive behaviors. Sensory hypo- or hyper-responsiveness has been frequently reported in individuals with neurodevelopmental disorders such as autism [16]. This includes unusual responses to visual stimuli, as documented by Simmons et al. [16], which have been suggested to explain the challenges in communication and social interactions in autism spectrum disorder (ASD). As the two siblings were previously diagnosed with childhood emotional disturbances, the hyperreactivity of their visual system might be related to hypervigilance and emotional regulation, similar to what is observed in individuals with autistic traits.

**Figure 7:**
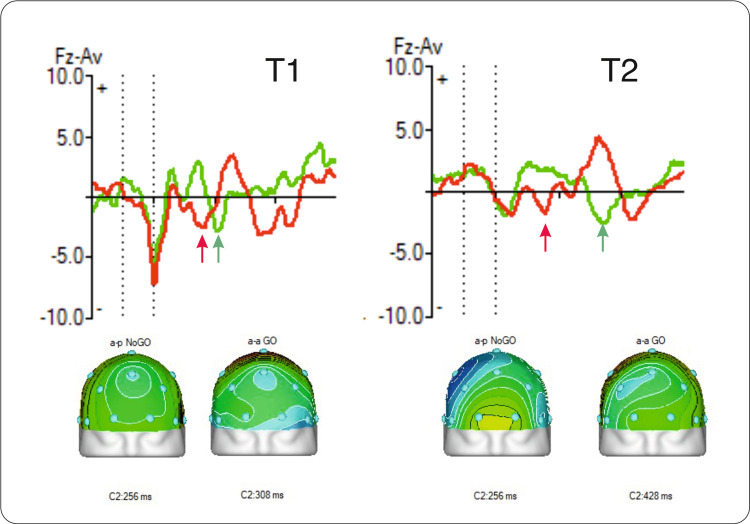
Similarities in N2 peak latencies in Go (green) and NoGo (red) trials in twin pair Arrows indicate peak latencies and maps show the location and time of the component peak (twins are coded as T1 and T2).

Furthermore, the maltreated twin pair exhibited similarities in the later cognitive component, N2 NoGo. The N2 component is associated with the maturation of the anterior cingulate cortex (ACC) and the development of the monitoring process [17]. As a component of motor inhibition, it has been found to be delayed in maltreated adolescents [18]. Early life stress was previously linked to a notable decrease in the amplitude of the N2 component [19]. Likewise, both twins (T1 and T2) also exhibit a reduction in the amplitude of this component, but the difference is not statistically significant compared to controls (p<0.3 in T1 and p<0.25 in T2). An interesting finding was the difference in N2 peak latencies between the Go and NoGo trials. Typically developed children have a faster N2 peak latency in Go trials compared to NoGo trials. In the maltreated twin pair, the N2 peak latency was shorter for the NoGo trials in both adolescents (Figure 7). Also, the “Go/NoGo N2 effect”, which is described as NoGo N2 being more negative, doesn’t show in this case.

The cognitive function for response inhibition was described in an "accumulation" model, which uses two independent "accumulators" that compete in building up neuronal activity for both initiating and stopping actions [13]. This competitive process was proposed as a potential neuronal substrate for response inhibition. To understand the structure of these "accumulators" in maltreated adolescents, we explored the cognitive latent components of ERPs, described by Kropotov [13], as indices for executive processes.

The latent ERP components that can be obtained with the independent component analysis (ICA) decomposition of ERPs exhibit specific temporospatial patterns and functional meanings [13]. Analyzing the activation pattern of latent ERP components for the N2 and P3 waves in the NoGo condition provides a more complex view of cognitive functioning impairments. Moreover, ICs of ERPs are suggested as better endophenotypes than raw ERPs [13], whose impairment might be more precisely associated with different brain dysfunctions. WinEEG software (ver. 3.13.26; Mitsar Co., Ltd., Saint Petersburg, Russia) was used to extract latent ICs through blind source separation, based on the second-order statistics method developed by Kropotov [13]. The procedure was adapted for ERPs, and the functional meanings of latent components were hypothesized in extensive work on functional neuromarkers in psychiatry [13]. IC topographies were extracted with the free sLORETA software from the Key Institute for Brain-Mind Research in Zurich, Switzerland.

IC1 (P1/N1): occipital-visual processing

**Figure 8:**
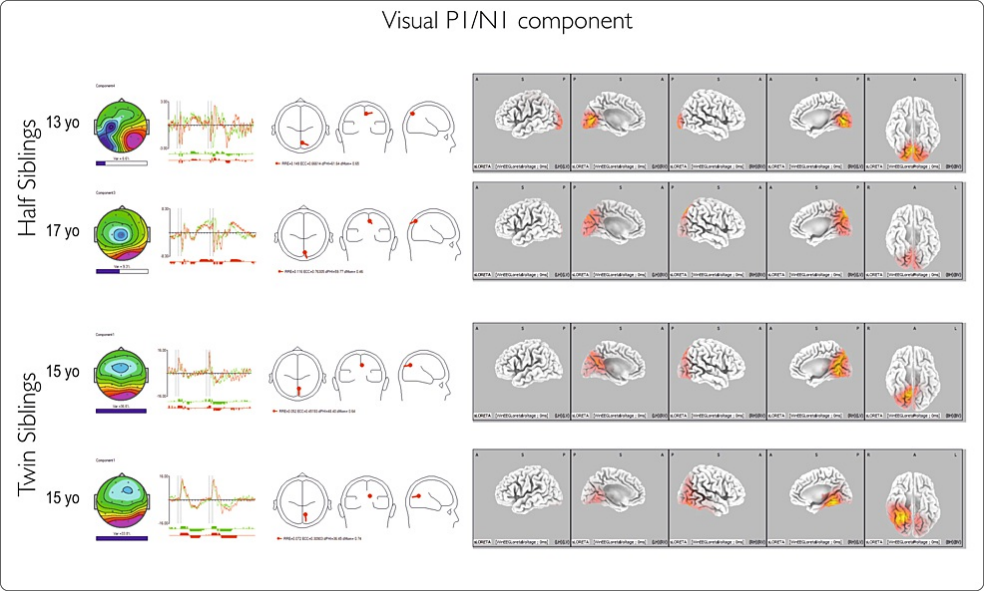
Latent visual component for Go and NoGo conditions Components are described (left to right) by (1) topography with the horizontal bar indicating the relative power of the component; (2) corresponding time courses for Go and NoGo conditions in 2000ms intervals; (3) dipole diagram; and (4) sLORETA image of the component. The first two rows represent occipital IC for half-siblings, and the last two rows are for twins.

The amplitude of this early component differs significantly from the norm for the two half-siblings but not for the twins. The much larger amplitude exhibited by the half-siblings is consistent with the large P1N1 ERP waves recorded in this pair, and it likely reflects an intensification of processing in primary and secondary visual cortical areas. The four cases show no linear relationship with age. The time course and topography of the ICs (Figure 8) share features with the conventional visual P1/N1 waves associated with visual recognition. The relative power of the component in the ERP wave exhibits high variability between the two pairs; twins show more than 30% weight of this component, whereas half-siblings show only 6-10%. The topography and dipole locations are similar for the four cases, with the twins showing a slight right lateralization.

IC2 (P3a): action suppression

**Figure 9:**
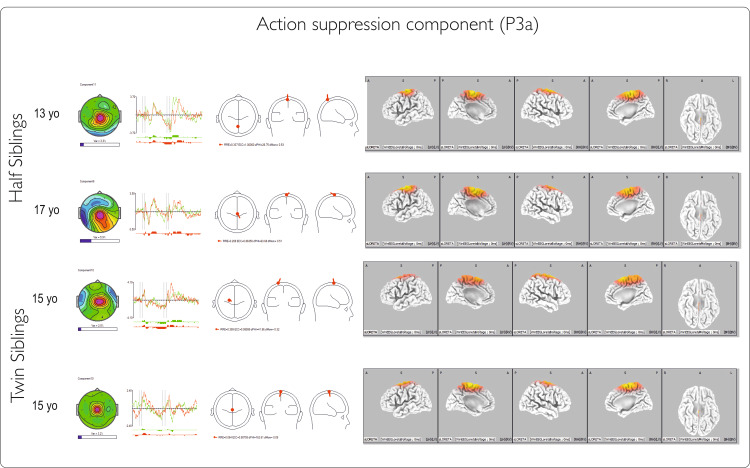
Action suppression component for Go and NoGo conditions Components are described (left to right) by (1) topography with the horizontal bar indicating the relative power of the component; (2) corresponding time courses for Go and NoGo conditions in 2000ms intervals; (3) dipole diagram; and (4) sLORETA image of the component. The first two rows represent IC for twin siblings, and the last two are for half-siblings. IC: independent component

This component is thought to be generated in the supplementary motor cortex [13], and its attributes relate to the N2 wave. As a functional meaning, the component reflects the operation of inhibiting the prepared action and was correlated with the neuropsychological domain of energization. The component exhibits early and main potentials. The latencies of the components are similar between pairs and faster for the half-sibling for the early potentials. For the main potentials, the latencies vary both within and between pairs. The component topography (Figure 9) is consistent in all four cases, with a similar area of generators and central localization. The relative power of this IC is low in all four participants.

IC3 (P4 monCC): conflict detection and monitoring

**Figure 10:**
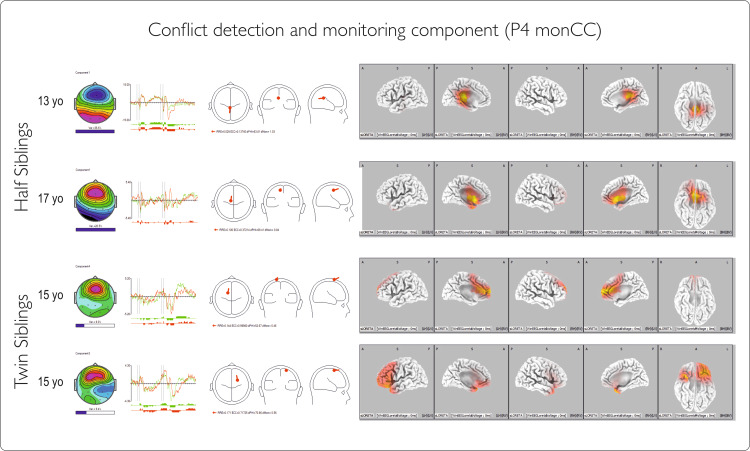
Conflict detection and monitoring component for Go and NoGo conditions Components are described (left to right) by (1) topography with the horizontal bar indicating the relative power of the component;(2) corresponding time courses for Go and NoGo conditions in 2000ms intervals; (3) dipole diagram; and (4) sLORETA image of the component. The first two rows represent IC for half-siblings, and the last two are for twins. IC: independent component

This component is generated in the anterior cingulate in adults and may have a less precise location of generators in children and adolescents [17]. The component revealed a main fluctuation at a similar latency for the twins and variable latency for the half-siblings, with large differences in relative power in the half-sibling pair compared with the twin pair. The sLORETA image shows that the activation pattern of the component becomes more frontal in late adolescents (S4, age 17) and has fewer generators in the cingulate in younger adolescents (S1, T1, T2, ages 13 and 15), with a more diffuse cortical distribution and even an inverse direction of the dipole in younger age (Figure 10). This may reflect the importance of the brain developmental stage in evaluating trauma-related neurophysiological changes.

**Figure 11:**
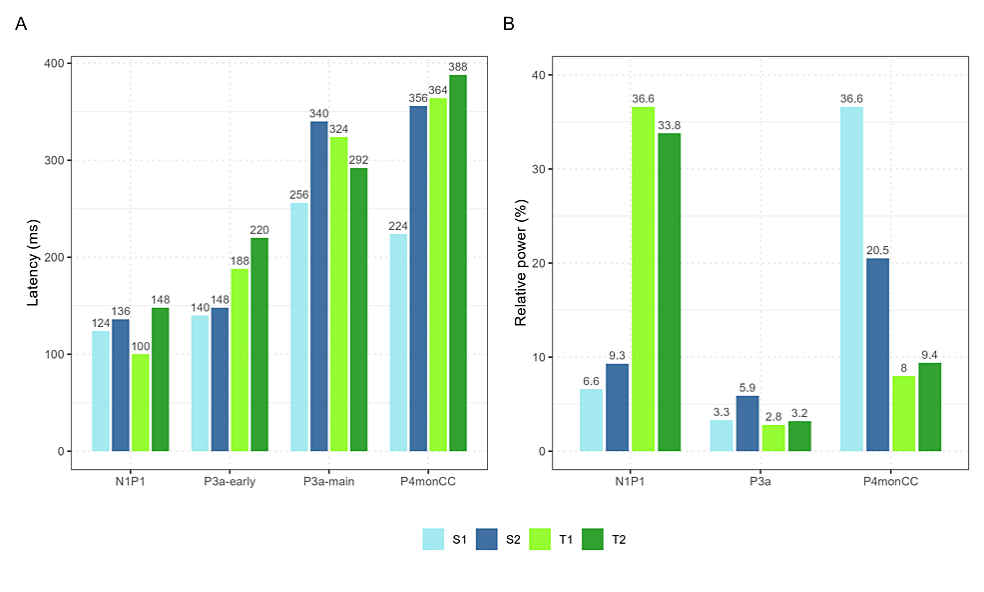
Comparative graphs of ICs latencies and relative power Panel A: ERP independent components latencies; Panel B: ERP independent components relative power. Twins = green, half-siblings = blue ICs: independent components; ERP: event-related potential

Early latent ERP components show no clear differences between pairs, while the later ones (P3a-main and P4monCC) have similar latencies for twins but not for half-siblings (Figure 11). This variation might be age-related, as the frontal cortex and cingulate are still developing. This is supported by graphical data showing later main inhibition and monitoring components for the older sibling.

## Discussion

We examined four cases of maltreatment in adolescents and identified subtle differences in the neurophysiological responses that may be linked to complex traumatic experiences. During a visual cognitive performance task, we observed decreased alpha power in the posterior region of the brain in individuals who had experienced physical abuse and bullying. We also found similar patterns of frontal midline theta distribution in twins, good behavioral performance across all participants (measured by errors and reaction time), and variances in some ERP components. Specifically, during cognitive control trials (NoGo condition), we noticed significantly higher amplitudes of the N1/P1 complex in half-siblings than in database norms, as well as lower power of the N2 in all four adolescents, although the latter was not statistically significant when compared to controls from the database. Similarities of theta scalp activation patterns (especially in the range of 7-8 Hz) and in NoGo N2 component peak and latency for the twin pair are consistent with the findings of Harper et al. [20]. They suggested that increased theta and delta activity elicited during the Go/NoGo task may index separate processes of response inhibition and may be uniquely related to N2 and P3 components.

Our results show altered neural responses during response inhibition in maltreated adolescents, supporting the view that executive functioning needs to be considered when dealing with such populations, especially when Axis I diagnostic criteria are not met (see [21]). Attention, working memory, and impulse control are considered the most affected by trauma, according to current literature [5]. However, the four participants exhibited no overall shared similarities in ERP components, except when compared in pairs, focusing on either their genetic background or their exposure to similar adverse events. Particularly, twins exhibited similarities in N2, while half-siblings shared similarities in N1/P1. Also, the two cases that were exposed to physical abuse and bullying (S2 and T2) had reduced N2 amplitude, and reduced alpha power in posterior locations, suggesting that this particular type of abuse may have more severe consequences on brain processing.

Our findings suggest that the N2 NoGo changes could be a promising marker that future research may address on larger samples to confirm its reliability and validity. If these findings are replicated, this marker, in conjunction with other trauma-related markers, could form the basis of a more precise diagnostic or classification algorithm. Considering that trauma is frequently misdiagnosed or underdiagnosed, utilizing a combination of such neural markers could be highly beneficial in providing more accurate diagnoses and appropriate interventions.

Our case analyses corroborate past research, showing that during adolescence and following exposure to complex trauma, some risk factors for PTSD, CPTSD, and other mental health issues are elicited. These risk factors include impairments in executive functioning that could be detected with central electrophysiological measures, especially attention and inhibition-related components (N1/P1 and N2). These impairments can be associated with genetic factors [15] or with the type of trauma experienced, with physical abuse being an exacerbating factor in altering neural responses. Moreover, our findings suggest that genes shared between individuals may be linked to common vulnerabilities in various stages of the information-processing circuit in the developing brain.

The current case study presents the descriptive results of an investigation into attentional processes in siblings raised in deprived environments, which includes sensory and cognitive processing systems. We found changes in some components of these systems when compared to the database, but not all of them were significant. These changes may indicate areas that are vulnerable to impairment, and could represent early indicators of the impact of adversities. If this is the case, replicating our results in a larger sample may contribute to understanding the timeline of trauma impact and provide opportunities for early intervention targeting the activation of crucial brain circuits related to cognitive control.

## Conclusions

The present study is a preliminary step toward a more comprehensive approach (brain and behavior) for CCT. It provides a broader view of particular cases that can complement cohort studies in the effort to understand the aftermath of trauma in developmental samples. Assessing trauma exposure in adults can be difficult, yet even more challenging in children and adolescents. Maltreated teenagers may have limited access to their internal psychological states, due to their age and the trauma they have experienced or still experience. Therefore, in addition to psychological assessment tools, biological measures are needed to make inferences about affected cognitive or emotional processes. This can help identify potential risk factors for further developing psychopathology. The key finding of this study is that even in a traumatic environment, the brain continues to develop, with the prefrontal cortex maturing and the main networks being constructed. For instance, in all four cases of maltreated adolescents, the latent components that generate orienting, monitoring, or inhibition response are present and located in their specific areas, while some subtle changes are observed in ERPs and qEEG metrics. Therefore, an important conclusion is that maltreatment may not cause irreversible damage at the age of adolescence. It does increase vulnerabilities, but there might still be a good chance of achieving positive results with interventions. Study results support the idea of an age-related window of opportunity for interventions to recover proper brain functioning. While a much larger sample will be needed to validate both qEEG metrics and ERP components that we reported as altered in the four cases we analyzed, the present case study may serve as an argument in favor of developing EEG-based measures to complement current screening tools, like clinical assessments and subjective reports. More generally, we emphasize that research at the intersection between clinical psychology and cognitive neuroscience is needed to further understand the interplay between neural processes and childhood trauma and to better evaluate cognitive abilities for treatment implementation.
